# Case Report: Abnormally Low Glycosylated Hemoglobin A1c Caused by Clinically Silent Rare β-Thalassemia in a Tujia Chinese Woman

**DOI:** 10.3389/fendo.2022.878680

**Published:** 2022-05-04

**Authors:** Wei Gao, Yanwen Jin, Minjin Wang, Yan Huang, Huairong Tang

**Affiliations:** ^1^ Health Management Center, West China Hospital, Sichuan University, Chengdu, China; ^2^ Biliary Surgery, West China Hospital, Sichuan University, Chengdu, China; ^3^ Department of Laboratory Medicine, West China Hospital, Sichuan University, Chengdu, China

**Keywords:** HbA1c, β-thalassemia, hemoglobinopathy, high-pressure liquid chromatography (HPLC), Hb J-Bangkok

## Abstract

**Background:**

Glycosylated hemoglobin A1c (HbA1c) is an important means of monitoring blood glucose and diagnosing diabetes. High-performance liquid chromatography (HPLC) is the most widely used method to detect HbA1c in clinical practice. However, the results of HbA1c by HPLC are susceptible to hemoglobinopathy. Here, we report a case of discordantly low HbA1c with an abnormal chromatogram caused by rare β-thalassemia.

**Case Description:**

A 36-year-old Tujia Chinese woman presented with an abnormally low HbA1c level of 3.4% by HPLC in a health check-up. The chromatogram of HbA1c showed an abnormal peak. Fasting blood glucose, routine blood tests and serum bilirubin were normal. Her body mass index was 27.86 kg/m^2^. Hemoglobin electrophoresis showed low hemoglobin A and abnormal hemoglobin β-chain variants. The thalassemia gene test suggested a rare type of β-thalassemia (gene sequencing HBB: c.170G>A, Hb J-Bangkok (GGC->GAC at codon 56) in a beta heterozygous mutation). Glycated albumin (GA) was slightly increased. Oral glucose tolerance tests (OGTT) and insulin release tests indicated impaired glucose tolerance and insulin resistance. The hematologist advised follow-up visits. The endocrinologist recommended that the patient adopt lifestyle intervention. Three months later, GA returned to normal, and impaired glucose tolerance and insulin resistance improved.

**Conclusions:**

Clinically silent β-thalassemia may lead to low HbA1c values and abnormal chromatograms by HPLC. In these circumstances, differential diagnosis is important. Checking the chromatogram may be helpful in interpreting HbA1c as well as identifying hemoglobinopathy. Further tests, such as GA, OGTT, hemoglobin electrophoresis and genetic tests, are needed for differential diagnosis.

## Background

Glycosylated hemoglobin A1c (HbA1c) is an important means of monitoring blood glucose for patients with diabetes (1, 2). The American Diabetes Association has listed HbA1c≥6.5% as a diagnostic criterion for diabetes since 2010 (3). High-performance liquid chromatography (HPLC) is more commonly used in the laboratory for the detection of HbA1c (4). However, the results of HbA1c by HPLC are susceptible to hemoglobinopathy (4). Here, we report a case of abnormally low HbA1c with an abnormal chromatogram caused by rare β-thalassemia.

## Case Description

A 36-year-old Tujia Chinese woman presented with an abnormally low HbA1c level of 3.4% in a health check-up. The fasting blood glucose level was 100.8 mg/dL (normal range 70.2-106.2). She did not have any complaints, past medical history or psychosocial history. Her mother was diagnosed with diabetes still on treatment. Physical examination showed that vital signs were stable. Cardiopulmonary examination showed no obvious abnormalities. There was no yellow staining of the skin or sclera. Other results were as follows: hemoglobin 138 g/L (normal range 115-150), red blood cell count 4.4*10^12^/L (normal range 3.8-5.1), mean corpuscular volume 96 fL (normal range 82-100), mean corpuscular hemoglobin 31 pg (normal range 27-34), mean corpuscular hemoglobin concentration 325 g/L (normal range 316-354), and serum bilirubin in the normal range. The results of laboratory examination are shown in **Table 1**. Her body mass index (BMI) was 27.86 kg/m^2^.

**Table 1:** Laboratory results of the patient.

Factors	Results	Reference range
HbA1c (%)	3.4	3.9-6.1
Fasting blood glucose (mg/dl)	100.8	70.2-106.2
Red blood cell count (*10^12^/L)	4.4	3.8-5.1
Hb (g/L)	138	115-150
MCV (fL)	96	82-100
MCH (pg)	31	27-34
MCHC (g/L)	325	316-354
Total bilirubin (µmol/L)	14.2	5.5-28.8
Direct bilirubin (µmol/L)	5.3	<8.8
Indirect bilirubin (µmol/L)	9.0	<20
Fasting plasma glucose in OGTT (mg/dl)	104.4	70.2-106.2
2 h plasma glucose (mg/dl)	166.3	59.4-140.4
Fasting insulin (uU/mL)	18.2	1.5-15.0
2 h insulin (uU/mL)	82.7	3.0-60.0
GA (%)	14.89	9-14
Fasting plasma glucose in OGTT 3months later (mg/dl)	90.5	70.2-106.2
2 h plasma glucose 3months later (mg/dl)	130.3	59.4-140.4
Fasting insulin 3months later (uU/mL)	10.5	1.5-15.0
2 h insulin 3months later (uU/mL)	56.1	3.0-60.0
GA 3months later (%)	13.67	9-14

**Figure 1:**
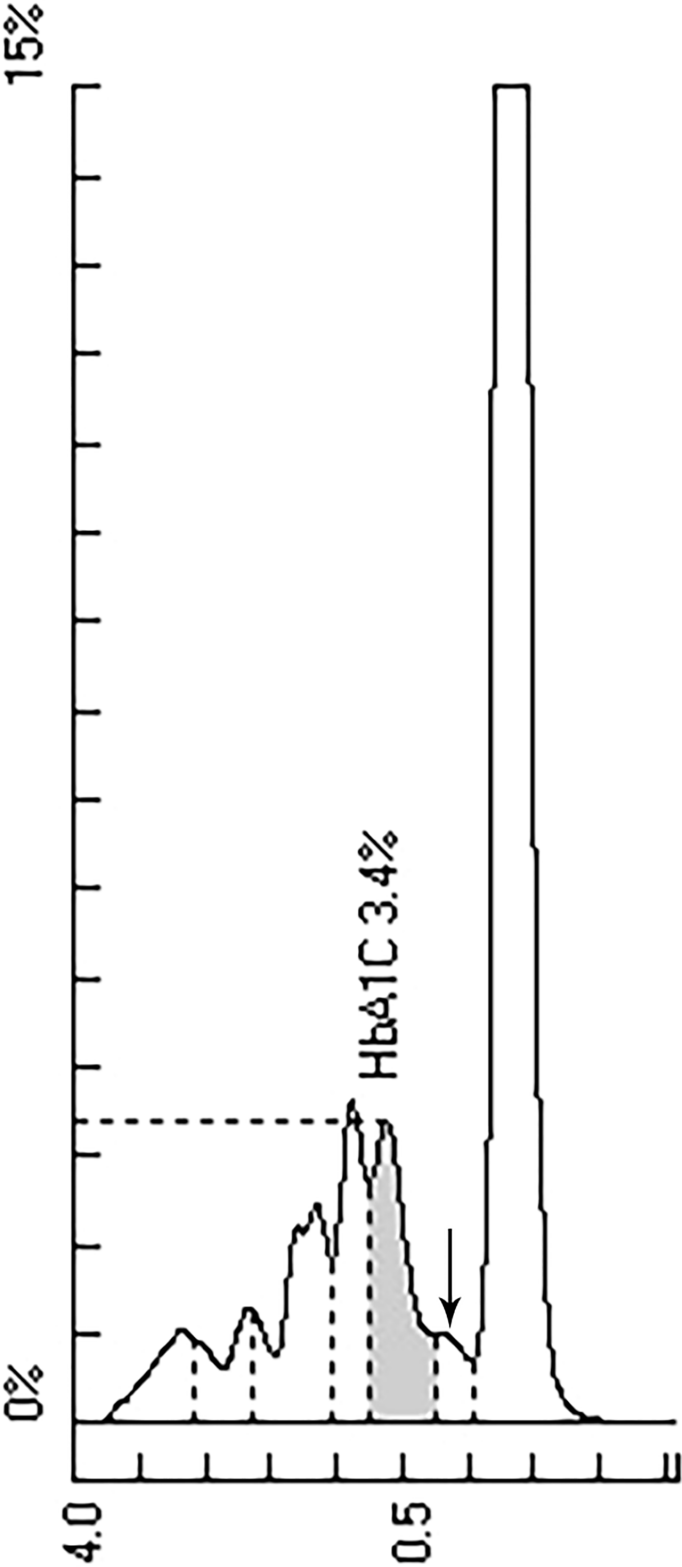
HPLC chromatogram of HbA1c. Abnormal peak marked by arrow.

**Figure 2:**
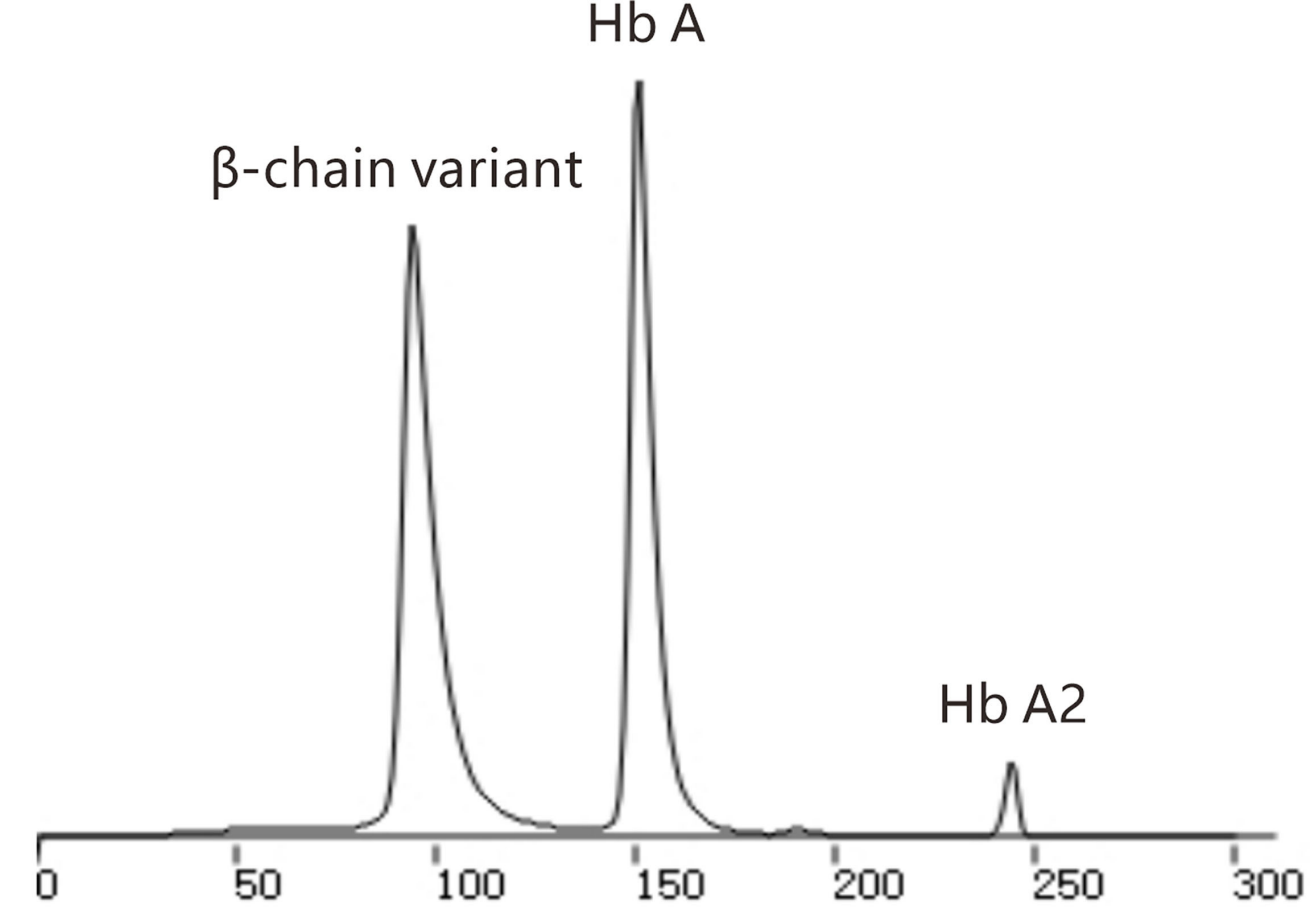
Chromatography of hemoglobin electrophoresis.

**Figure 3:**
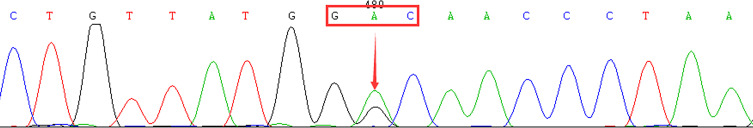
Gene mutation in high-throughput sequencing.

HbA1c was measured by ion exchange HPLC (TOSOH HLC-723G11). The chromatogram showed an abnormal peak between A1c and A0 (**Figure 1**). The primary care physician prescribed further tests after full communication with the patient. Hemoglobin electrophoresis indicated that hemoglobin A accounted for 45.4% (normal range 96-97.6), and abnormal hemoglobin β-chain variants accounted for 52.1% (**Figure 2**). The thalassemia gene test suggested a rare type of β-thalassemia (gene sequencing HBB: c.170G>A, Hb J-Bangkok (GGC->GAC at codon 56) in a beta heterozygous mutation) (**Figure 3**), which was not one of the 17 common β-thalassemia-related gene point mutations in Chinese individuals. Glycated albumin (GA) was 14.89% (normal range 9-14%). The results of the oral glucose tolerance test (OGTT) and insulin release test were as follows: fasting plasma glucose 104.4 mg/dL (normal range 70.2-106.2), 2 h blood glucose 166.3 mg/dL (normal range 59.4-140.4), fasting insulin 18.2 µU/mL (normal range 1.5-15.0) and 2 h insulin 82.7 µU/mL (3.0-60.0). The patient was diagnosed with prediabetes.

Due to a lack of clinical manifestations and normal routine blood results, the hematologist recommended follow-up visits. The patient had impaired glucose tolerance and insulin resistance, and the endocrinologist recommended that the patient adopt lifestyle intervention, mainly including dietary intervention and exercise intervention. The patient readily consented to the treatment. Patient compliance was ensured by monthly visits or telephone follow-up. The adherence and tolerability of lifestyle intervention in this patient were very good, and there were no adverse or unanticipated events. Three months later, BMI was 26.12 kg/m^2^, GA was 13.67% (normal range 9-14%), and the results of the OGTT and insulin release test were as follows: fasting plasma glucose 90.5 mg/dL (normal range 70.2-106.2), 2 h blood glucose 130.3 mg/dL (normal range 59.4-140.4), fasting insulin 10.5 µU/mL (normal range 1.5-15.0) and 2 h insulin 56.1 µU/mL (3.0-60.0). Impaired glucose tolerance and insulin resistance improved. After that, the patient was followed up every 3 months.

## Discussion

HbA1c is the product of a nonenzymatic reaction between hemoglobin and serum glucose. The nonenzymatic reaction is persistent, slow and irreversible. HbA1c is generally considered to be a useful indicator of average blood glucose levels over the past 8 to 12 weeks. HbA1c is an important means of monitoring blood glucose and diagnosing diabetes (1–3).

Hemoglobinopathy is a group of genetic disorders, including abnormal hemoglobin disease and thalassemia, which is one of the influencing factors for HbA1c detection (5, 6). The incidence of hemoglobinopathy is high in Sichuan Province, China (7, 8). Previous studies have suggested that mild β-thalassemia has no significant effect on HbA1c (9), but the HbA1c result of this patient appears to be much lower despite the absence of anemia and hemolysis. We speculate that there are two reasons for the low HbA1c. First, hemoglobin A had a low proportion, leading to the discordantly low HbA1c in this patient. The second cause is the presence of Hb J-Bangkok in the β-globin gene sequencing. The principle of the ion exchange HPLC method is to separate HbA1c from Hb A based on charge difference (5). Hb J-Bangkok is a hemoglobin variant defined as GGC->GAC at codon 56 (10). The charge of Hb J-Bangkok changed with the change in amino acids, and Hb J-Bangkok migrated with HbA0 separating from HbA1c (5), which resulted in a decrease in the value of HbA1c. Even with clinical silence, the rare type of β-thalassemia (gene sequencing HBB: c.170G>A, Hb J-Bangkok (GGC->GAC at codon 56) in beta heterozygous mutation) can lower HbA1c values.

At present, HPLC, immunoassays, capillary electrophoresis and enzyme methods are used to determine HbA1c (11, 12). HPLC is the most widely used method to detect HbA1c in clinical practice, and the results are directly used by clinicians to evaluate the long-term control of blood glucose in diabetic patients (13, 14). The reason is that HPLC provides a chromatography figure for each patient sample, which makes it easy to interpret HbA1c (15). However, HPLC cannot eliminate the influence of abnormal hemoglobin on HbA1c (5, 16). Endocrine or primary care physicians should check chromatograms when HbA1c is discordantly high or low. When abnormal HbA1c values and/or chromatograms are found, further hemoglobin electrophoresis and genetic tests should be ordered to further exclude hemoglobinopathy after full communication with the patient. The accuracy of HbA1c in patients with hemoglobinopathy is method dependent. It has been reported that immunoassay and enzyme methods are not affected by Hb J-Bangkok (17). Immunoassays use specific antibodies to bind to glycated sites on the N-terminus of the Hb β chain of HbA1c, which is far from the location of the amino acid substitution of Hb J-Bangkok. Therefore, Hb J-Bangkok does not affect antibody recognition (17). The principle of enzymatic determination of HbA1c is that glycated glycine glutamine from the β chain N-terminus of HbAlc is cut off by a specific protease. The specific cleavage site of HbA1c is not affected by Hb J-Bangkok (17).

If other methods for determining HbA1c are not available in the laboratory, GA, OGTT, blood glucose self-monitoring and dynamic blood glucose monitoring are also alternative options. GA is the second indicator to evaluate the level of average blood glucose and is not unaffected by abnormal hemoglobin (18). In areas with a high prevalence of hemoglobinopathy, the role of GA must be emphasized in blood glucose control assessment and diabetes screening (19). This patient had an abnormal HbA1c value and chromatogram, and further examination revealed impaired glucose tolerance as well as insulin resistance. She was relieved after timely lifestyle intervention. Prediabetes would be a missed diagnosis solely based on the HbA1c value and fasting blood glucose in this patient.

## Conclusion

Clinically silent β-thalassemia may lead to low HbA1c values and abnormal chromatograms by HPLC. In these circumstances, differential diagnosis is important. Checking the chromatogram may be helpful in interpreting HbA1c as well as identifying hemoglobinopathy. Further tests, such as GA, OGTT, hemoglobin electrophoresis and genetic tests, are needed for differential diagnosis.

## Data Availability Statement

The original contributions presented in the study are included in the article/**Supplementary Material**. Further inquiries can be directed to the corresponding author.

## Ethics Statement

Written informed consent was obtained from the individual(s) for the publication of any potentially identifiable images or data included in this article.

## Author Contributions

WG wrote the manuscript. YJ and YH reviewed the manuscript. MW interpreted the laboratory results. HT was responsible for the study design and manuscript revision. All authors contributed to the article and approved the submitted version.

## Funding

This study was supported by Scientific and technological Achievements Transformation Fund of West China Hospital, Sichuan University (CGZH19013), Science and Technology Bureau of Sichuan Province (grant numbers: 2020YFS0099, 2021YJ0139, 2019YFS0306).

## Conflict of Interest

The authors declare that the research was conducted in the absence of any commercial or financial relationships that could be construed as a potential conflict of interest.

## Publisher’s Note

All claims expressed in this article are solely those of the authors and do not necessarily represent those of their affiliated organizations, or those of the publisher, the editors and the reviewers. Any product that may be evaluated in this article, or claim that may be made by its manufacturer, is not guaranteed or endorsed by the publisher.
