# Supplementary material for: Case Report: Abnormally Low Glycosylated Hemoglobin A1c Caused by Clinically Silent Rare β-Thalassemia in a Tujia Chinese Woman
Source: Front Endocrinol (Lausanne). 2022 May 4;13:878680. doi: 10.3389/fendo.2022.878680 (PMC9114733; doi:10.3389/fendo.2022.878680)
Supplement: Supplementary file 1 [file Table_1.docx]

**TABLE 1. Laboratory results of the patient**

| Factors | Results | Reference range |
| --- | --- | --- |
| HbA1c (%) | 3.4 | 3.9-6.1 |
| Fasting blood glucose (mg/dl) | 100.8 | 70.2-106.2 |
| Red blood cell count (*10^12^/L) | 4.4 | 3.8-5.1 |
| Hb (g/L) | 138 | 115-150 |
| MCV (fL) | 96 | 82-100 |
| MCH (pg) | 31 | 27-34 |
| MCHC (g/L) | 325 | 316-354 |
| Total bilirubin (µmol/L) | 14.3 | 5.5-28.8 |
| Direct bilirubin (µmol/L) | 5.3 | <8.8 |
| Indirect bilirubin (µmol/L) | 9.0 | <20 |
| Fasting plasma glucose in OGTT (mg/dl) | 104.4 | 70.2-106.2 |
| 2 h plasma glucose (mg/dl) | 166.3 | 59.4-140.4 |
| Fasting insulin (uU/mL) | 18.2 | 1.5-15.0 |
| 2 h insulin (uU/mL) | 82.7 | 3.0-60.0 |
| GA (%) | 14.89 | 9-14 |
| Fasting plasma glucose in OGTT 3months later (mg/dl) | 90.5 | 70.2-106.2 |
| 2 h plasma glucose 3months later (mg/dl) | 130.3 | 59.4-140.4 |
| Fasting insulin 3months later (uU/mL) | 10.5 | 1.5-15.0 |
| 2 h insulin 3months later (uU/mL) | 56.1 | 3.0-60.0 |
| GA 3months later (%) | 13.67 | 9-14 |
